# Clinical and Radiological Outcomes after Knee Arthroplasty with Patient-Specific versus Off-the-Shelf Knee Implants: A Systematic Review

**DOI:** 10.3390/jpm11070590

**Published:** 2021-06-22

**Authors:** Céline Saphena Moret, Benjamin Luca Schelker, Michael Tobias Hirschmann

**Affiliations:** 1 Department of Orthopaedic Surgery and Traumatology, Kantonsspital Baselland (Bruderholz, Liestal, Laufen), CH-4101 Bruderholz, Switzerland; celinesaphena.moret@unibas.ch (C.S.M.); benjamin.schelker@unibas.ch (B.L.S.); 2 Department of Clinical Research, Research Group Michael T. Hirschmann, Regenerative Medicine & Biomechanics, University of Basel, CH-4001 Basel, Switzerland

**Keywords:** total knee arthroplasty, customised, patient specific, personalised, knee replacement

## Abstract

Customised, patient-specific implants (PSI) manufactured based on computed tomography data are intended to improve the clinical outcome by restoring more natural knee kinematics as well as providing a better fit and a more precise positioning. The aim of this systematic review is to investigate the effect of these PSI on the clinical and radiological outcome compared to standard, off-the-shelf (OTS) implants. Thirteen comparative studies including a total of 2127 knee implants were identified. No significant differences in clinical outcome assessed with the range of motion, the Knee Society Score (KSS), and the Forgotten Joint Score (FJS-12) were found between PSI and OTS implants. PSI showed fewer outliers from the neutral limb axis and a better implant fit and positioning. Whether these radiological differences lead to long-term advantages in terms of implant survival cannot be answered based on the current data. Patients receiving PSI could be discharged home earlier at the same or at an even lower total cost. The effective overall superiority of PSI has yet to be proven in long-term studies.

## 1. Introduction

Total knee arthroplasty (TKA) is a successful and effective treatment for end-stage knee osteoarthritis (OA) [1,2]. With the increase in life expectancy and in the prevalence of obesity, OA has become a relevant cause of disability worldwide, thus leading to a rise in the number of TKA performed [3,4]. However, up to 20% of patients are dissatisfied with the clinical outcome of the surgery as they suffer from persistent pain, instability, persistent or recurrent effusion, and limited knee function [5,6,7,8,9]. The possible reasons for these unsatisfactory outcomes are manifold and often lead to revision arthroplasty. In particular, aseptic loosening, instability, and patellofemoral disorders, which are responsible for about 40% of all revision causes, are known to be affected by the size or positioning of the implant [10,11,12]. A potentially relevant approach to improve the outcome after knee arthroplasty, which besides enhancing surgical precision and defining an optimal alignment strategy, consists of developing new implant designs. Conventional, off-the-shelf (OTS) implants were developed on the basis of anthropometric measurements of a defined standard population [13]. Although different models and sizes of OTS implants exist, it can be challenging to find the best fitting implant design and size for the individual patient’s knee morphology. In addition, the choice of implant is also limited by the surgeon’s preferences and experience with different models or the availability in a particular hospital. Modern imaging and implant fabrication techniques make it possible to produce patient-specific instrumentation and implants in order to better fit the individual anthropometric knee joint morphology. The crucial question is whether patients benefit from a more individualised approach using patient-specific implants (PSI). Hence, the aim of this systematic review is to (1) compare clinical outcomes of patient-specific unicompartmental knee arthroplasty (UKA) and TKA implants (PSI) with OTS implants, (2) investigate the radiological outcome such as the implant and limb alignment, and (3) examine the impact of individualised implants on procedure-related factors such as cost, length of hospital stay, discharge destination, and blood loss.

## 2. Materials and Methods

### 2.1. Search Strategy

A systematic literature search was conducted on PubMed, Medline, Embase, Cochrane, Scopus, and World of Science from their inception until 5 March 2021 to identify potentially relevant articles for this review. Terms including “unicondylar knee replacement”, “unicondylar knee arthroplasty”, “unicondylar knee prosthesis”, “partial knee replacement”, “partial knee arthroplasty”, “unicompartmental knee replacement”, “unicompartmental knee arthroplasty”, “unicompartmental knee prosthesis”, “UKA”, “total knee replacement”, “total knee arthroplasty”, “total knee prosthesis”, “TKA”, “patient-specific”, custom*, “individually made”, “off-the-shelf”, commercial*, and convention* were searched for in both the title and abstract.

Inclusion criteria comprised publications in English or German in peer-reviewed journals comparing patient-specific with standard implants. Only full-text articles were included. Following the compilation of all identified articles and removal of duplicates, two investigators (BLS, CSM) independently screened the studies for inclusion criteria by title and abstract. Then, selected articles were scanned by full text on their eligibility. In case of discrepancies, a third author was consulted (MTH). In addition, manual screening of the reference lists of articles that met the above-mentioned criteria was conducted for additional studies that were not covered by the original search terms.

For this systematic review, only studies comparing clinical outcomes with validated assessment methods or clear endpoints between PSI and OTS implants for UKA and TKA were included. These outcomes contained the Knee Society Score (KSS) [14], specific patient-reported outcome measures (PROMs), the range of motion (ROM), and radiological measurements as well as manipulation under anaesthesia (MUA) and revision rates. Further studies assessing procedure-related factors such as costs, length of hospital stay, discharge destination, and blood loss were also included. All prospective trials and retrospective studies were considered.

Articles regarding patient-specific knee implants for complex bony reconstructions or tumour surgery and patient-specific instrumentation solely (without patient-specific implants) as well as simulation studies, review articles, case reports and editorial comments were excluded. 

### 2.2. Quality Assessment 

The methodological quality of the included studies and the risk of bias were assessed using the Methodological Index for Non-Randomised Studies (MINORS) for non-randomised comparative and non-comparative clinical intervention studies [15]. MINORS proposes a global ideal score of 16 for non-comparative studies and of 24 for comparative studies.

### 2.3. Data Extraction 

One of the authors (BLS) extracted the data from the selected publications into a Microsoft Excel spreadsheet. Then, the other author (CSM) checked the input for errors. The following information was extracted from the studies: title, author, year of publication, study design, level of evidence, number of knees in each study group, implant types, follow-up time, patient demographics, clinical outcome scores, revision rates, MUA rates, ROM, costs, hospitalisation time, discharge destination, blood loss, and radiological outcome measures. 

### 2.4. Statistical Analysis

Continuous variables were described with means and standard deviations or medians and ranges. Categorical variables were given with absolute and relative frequencies. Some of the results were only available as ranges and not as standard deviations (SD), limiting the comparability of the individual studies. Due to the great heterogeneity of the available studies, it was not possible to conduct a meta-analysis. For data interpretation, a *p* < 0.05 was considered statistically significant.

## 3. Results

### 3.1. Search Results and Characteristics of Included Studies 

**Figure 1:**
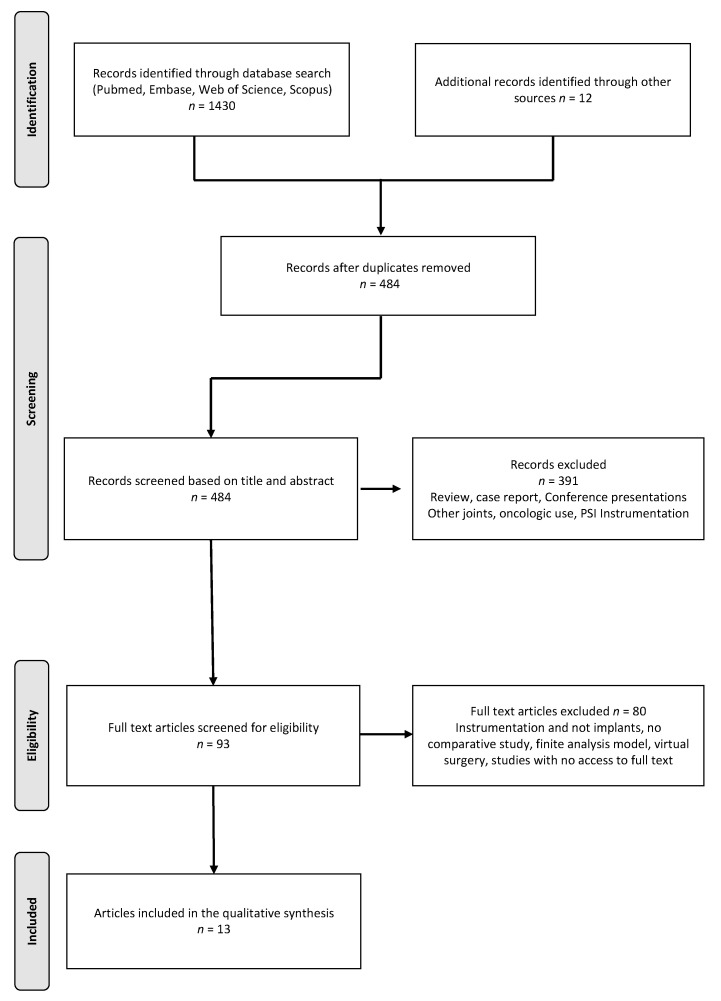
Flowchart of the study selection process according to the PRISMA Statement for the Conduct of Systematic Reviews.

The literature search yielded a total of 1430 publications and, after allocation processes shown in Figure 1, 13 articles met the criteria for this systematic review. Of these articles, 11 investigated the outcomes after TKA [16,17,18,19,20,21,22,23,24,25,26] and two investigated the outcomes after UKA [27,28] with PSI versus OTS implants. There were four prospective cohort studies and nine retrospective cohort studies. According to MINORS for comparative studies, the mean global score was 17.7 (SD ± 2). Further characteristics of the included studies are listed in Table 1. 

### 3.2. Patient Characteristics 

In this review, a total of 2127 knee implants were assessed. Of the these, 2034 and 93 underwent TKA and UKA, respectively. TKA patients received 1028 PSI systems and UKA patients received 53 PSI systems. In addition, O’Conner et al. [21] examined 4434 knees for the resulting costs only. Patient demographics of the included studies can be found in Table 2.

### 3.3. Implant Types

For TKA, ConforMIS’ first and second generation iTotal^®^ implants were used as PSI and compared to one or two different OTS implants (Table 2). In patients requiring a UKA, ConforMIS’ iUni^®^ implants were compared to OTS implants (Table 2).

### 3.4. Clinical Outcome 

White and Ranawat [26] asked patients to rate their satisfaction regarding their knee implant on a scale from 1 (unsatisfied) to 10 (fully satisfied). The OTS CR (mean 8.3, SD ± 2.2, *p* = 0.04) and OTS PS (mean 8.9, SD ± 1.0, *p* = 0.01) implant group reported significantly higher satisfaction than PSI (mean 7.0, SD ± 2.1). 

Buch et al. [17] found a significantly greater mean postoperative ROM in the PSI group compared to the OTS implant group (122° versus 114°, *p* < 0.001). In contrast, Schwarzkopf et al. [24] reported a decrease of 3.44° (range, −83° to 55°) in ROM after TKA with PSI, whereas patients receiving OTS implants showed an increase of 1.54° (range, −80° to 90°, *p* < 0.1). The remaining authors did not observe statistically significant differences in ROM between both groups [20,22,25,26,27].

With regard to the KSS, Wheatley et al. [25] only found a non-significant difference in both the knee score and the function score. Reimann et al. [22], on the other hand, found a significantly better function score in the PSI compared to the OTS implant group. White and Ranawat [26] determined a significantly lower the knee score in the PSI group (85.4 points) compared to both OTS implant groups (95.5 and 97.3 points), whereas Meheux et al. [20] found no significant differences. 

Wheatley et al. [25] also assessed the Forgotten Joint Score (FJS-12), which showed no significant difference between PSI and OTS implant groups. Furthermore, the Western Ontario and McMaster Universities Arthritis (WOMAC) questionnaire was conducted by White and Ranawat [26]. The OTS CR implant group showed a significantly better total score than the PSI group (*p* = 0.04). Further results regarding the clinical outcome are provided in Table 3.

### 3.5. Revisions and Reoperations

Looking at the rate of MUA, White and Ranawat [26] observed that six of the 21 (28.6%) patients in the PSI group required manipulation compared to none in the OTS implant group. However, these results were not replicated in the other studies, where the rate of MUA did not differ between both PSI and OTS implant groups [17,25]. In the study by Meheux et al. [20], the iTotal^®^ G2 (ConforMIS) system showed a revision rate of 23% (18/77) compared to 2.4% (1/41) for the OTS implant. This led the PSI system to be discontinued during the study period and exchanged for the iTotal^®^ G2 plus (ConforMIS) system. None of the patients subsequently operated on with the new system required revision within the two-year follow-up period. Wheatley et al. [25] reported four patients needing arthroscopic debridement due to retropatellar crepitations in the PSI group compared to one arthroscopic debridement in the OTS group. However, all but one of the included studies assessing revisions after TKA found higher revision rates in the OTS groups [17,22,27,28].

### 3.6. Radiological Outcomes

Comparing the frontal tibial component angle (FTC) of the implants to the target values of 90°, Meheux et al. [20] demonstrated that the PSI-1 and OTS implant groups deviated significantly from the target in contrast to the PSI-2 group. The study by Ivie et al. [19] could not confirm these results. However, the same authors [19] found a significant difference in the frontal femoral component angle (FFC) angle between OTS implants and PSI. Although the mean FFC was within the desired +3° of deviation from the neutral axis (90°) for both groups, the femoral component of the PSI was 1.5 times more likely to be within this desired range than that of the OTS implants. No further studies included in the review reported on the FFC (Table 4).

Ivie et al. [19] found a mean postoperative hip–knee–ankle angle (HKA) significantly closer to the neutral limb alignment in the PSI group (PSI, 0.47° of varus ± 3.15° versus OTS implants, 1.68° of valgus ± 3.65°; *p* ≤ 0.01). In contrast, Arbab et al. [16] and Meheux et al. [20] found no significant difference in the HKA between PSI and OTS implant cohorts. However, Arbab et al. [16] and Ivie et al. [19] reported fewer outliers from neutral alignment (±3°) in the PSI group compared to the OTS implant group. 

Schroeder et al. [23] investigated the fit of different types of tibial components intraoperatively. PSI achieved an optimal fit (i.e., ≤1 mm of overhang or undercoverage) or relative undercoverage of 1–3 mm in 80% of case in contrast to 27% for OTS implants (*p* < 0.001). Demange et al. [27], who investigated the optimal fit of UKA implants, found that 75.8% of PSI and 21.1% of OTS implants achieved of an optimal fit. 

The rotational alignment of the tibial component was also analysed by Schroeder et al. [23] using a computer-aided design (CAD) during a virtual surgery. When a maximal tibial bone coverage was opted for, the rotational alignment did not have to be compromised in the PSI group in contrary to OTS implant group, which showed a greater mean deviation from the adequate alignment.

### 3.7. Procedure-Related Factors

O’Connor et al. [21] attributed a statistically significant average savings of 1695 USD ($18,585 versus $20,280; <0.0001) in total costs to PSI. However, another author only found a non-significant differences in costs in favour of PSI (PSI $21,591 ± 4439 versus OTS $22,092 ± 5940) [18]. Significantly lower were also the costs for follow-up care in the PSI group ($5048 ± $2929 versus $6361 ± $4482; *p* = 0.007).

In terms of length of hospital stay, patients undergoing UKA with a PSI spent an average of 8.4 days (SD ± 1.5, *p* < 0.003) in hospital compared to 10.9 days (SD ± 2.9) with an OTS implant [28]. Similarly, a significantly shorter length of stay was calculated for TKA using PSI by Schwarzkopf et al. [24] (2.44 vs. 3.18, *p* < 0.01), Meheux et al. [20] (OTS vs. PSI 1 vs. PSI 2, 3.3 ± 1.2 vs. 2.88 ± 1.1 vs. 2.08 ± 0.6, *p* < 0.01) and Buch et al. [17] (OTS vs. PSI, 2.7 vs. 1.6, *p* = 0.004).

No significant differences were seen in the duration of surgery in both groups for UKA and TKA [24,28]. Buch et al. [17] found the proportion of patients discharged home to be significantly higher in the PSI group (97% versus 80%, *p* = 0.05), whereas Culler et al. [18] found no significant difference between groups. In addition, Meheux et al. [20] also recorded a lower postoperative haemoglobin (Hg) drop in the PSI 2 group compared to the OTS implant group (0.61 ± 0.3 vs. 1.20 ± 1.3, *p* < 0.05).

## 4. Discussion

The key question to be answered by this review is whether patients undergoing TKA or UKA with a PSI present a better clinical outcome than with OTS implants. Based on the results of the included studies, no clear advantage of PSI over OTS implants were identified. Nonetheless, the results of the included studies have proven the non-inferiority of PSI in terms of clinical outcomes compared to OTS implants.

Implications for decisive improvements in clinical outcome favouring PSI are drawn from promising results of kinematic and biomechanical studies as well as PROMs data from various case series [29,30,31,32]. For instance, Zeller et al. [33] howed that PSI have more normal and physiological kinematics corresponding to the native knee than OTS implants. Patil et al. [34] came to a similar conclusion based on the results of their cadaver study. Due to the lack of an OTS implant control group, case series regarding the clinical and radiological outcome of PSI were excluded from the present study [29,30,31,32]. 

In this study, only one publication addressed patient satisfaction [26]. However, the determined inferiority of PSI compared to OTS implants is inconsistent with the data presented by Katthagen et al. [35], which was not included in the present study due to the unavailability of the full text manuscript. In contrary to White and Ranawat [26], reporting an increased rate of MUA in the PSI group, more recent studies did not support those findings [25,36]. Hence, future studies should potentially take this aspect into account.

Considering the revision rate, most of the included studies reported lower revision rates in the PSI group [17,27,28]. However, no explanation could be found for the increased incidence of patellar crepitations, requiring arthroscopic debridement, in said group in the study by Wheatley et al. [25]. This complication was not described by the other authors.

The mechanical alignment most surgeons aim for still remains the standard alignment target. A postoperative limb alignment within ±3° from the neutral axis is generally considered a "safe zone", as studies by Ritter et al. [37] and Fang et al. [38] have shown that deviation from this range is associated with a higher failure rate and shorter implant survival. All included studies assessed the ConforMIS PSI, which applies the traditional mechanical alignment strategy. Indeed, two of these found that the proportion of outliers > 3° deviation from the neutral axis in the coronal plane were lower in the PSI group than in the OTS implant group [16,19]. This is consistent with the findings of a case series by Levengood et al. [39] and Arnholdt et al. [40]. Whether the more precise alignment is actually a result of the patient-specific implants or rather the patient-specific instrumentation is questionable [41]. Furthermore, it is debatable to what extent patients benefit from the apparent better mechanical alignment of the implants, as recent studies have shown no detrimental influence of varus and valgus outliers > 3° on implant survival after 10 and 20 years [42,43]. 

Indeed, the optimal realignment strategy is currently undergoing a paradigm shift away from a strict mechanical alignment and towards a more personalised alignment. Another PSI manufacturer Symbios (Yverdon-les-bains, Switzerland), which has not yet been included in comparative studies because of its quite recent entry on the market, applies a recently developed individualised alignment strategy. It is based on the restricted phenotype alignment, which allows a better reproduction of the patient-specific limb alignment in addition to the individual knee morphology [44]. Combining a patient-specific implant with a more individualised alignment strategy seems promising; however, long-term studies assessing the impact of this alignment on the clinical outcome are still lacking.

It is commonly accepted that the optimal rotational alignment of the implant components is crucial. Internal rotation of the tibial component has been shown to be associated with poorer clinical outcome and is considered a major cause of postoperative pain [45,46]. Schroeder et al. [23] simulated the compromise between adequate bone coverage and optimal rotation alignment that has to be made when using OTS tibial components, which is not the case with PSI due to their individualised design. Although intuitive, these results should be verified in comparative cohort studies on postoperative radiological exams. 

The improved tibial bone coverage of the PSI was demonstrated in several studies included in the review as well as in case series [23,27,47]. It has been shown that the anteroposterior to mediolateral femoral condyle ratios are related to ethnicity and gender [48,49]. The use of PSI in patients who present less conventional anthropometric characteristics is expected to reduce femoral component overhang and undercoverage as well as the associated increased risks of postoperative pain and functional limitations [50,51]. The better bone coverage and potentially shorter surgery time with PSI could be seen as the reason for the lower blood loss and Hb drop [18,24]. Other beneficial effects of an optimal tibial fit are a decreased risk of subsidence and soft tissue impingement [52]. Furthermore, PSI allow a more precise rotational alignment of the femoral component in addition to recreating the individual trochlear groove matching the shape of the patella. This improves patellar tracking by maintaining its native alignment. Nevertheless, this aspect has not yet been assessed in comparative studies; thus, no conclusions can be drawn in this regard.

With rising healthcare costs worldwide and an increase in patients requiring TKA, there is concern that providing patients with PSI will result in higher costs compared to OTS implants. PSI indeed have higher upfront costs due to the required preoperative imaging and the customised manufacturing process [53]. However, Culler et al. [18] saw no difference in overall costs, and O’Conner et al. [21] even found significantly lower costs in the PSI group when looking at total postoperative costs up to one year after surgery. Possible reasons for the lower total costs seem to be the reduced length of hospital stay and fewer discharge to rehabilitation facilities compared to OTS implants [17,18]. However, this has to be taken with a grain of salt, as patients receiving PSI tend to be younger, healthier, and of a higher socioeconomic status.

The most relevant limitation of this systematic review is the heterogenic radiological endpoints and outcome assessment methods used in the included studies, which rendered a comparison difficult. In addition, the quality of these studies was rather low with an average MINORS of 17.7 (SD ± 2) and only few authors performing a sample size power calculation beforehand. Due to the higher upfront cost, it is suspected that many of these TKA with PSI were performed in private hospitals or at least on patients with additional insurance, which may lead to a selection bias. Moreover, the TKA were performed in Western countries, with a probably mostly Caucasian population, although it is suspected that PSI could be especially beneficial for patient with different anthropometric measurement (i.e., ethnic backgrounds). Lastly, since PSI were first introduced to the market about a decade ago and many single cohort studies show promising results, long-term comparative studies are still lacking. However, a paradigm shift in the field of knee arthroplasty towards a more personalised approach that combines enhanced surgical accuracy using patient-specific instrumentation, individualised alignment strategies, improved fit with customised implants and thus a better restoration the native knee joint seems ineluctable.

## 5. Conclusions

This study demonstrates inconclusive results and mostly non-significant differences in terms of clinical outcome between PSI and OTS implants. Although the use of PSI resulted in a better alignment as well as implant fit and positioning, these improved radiological findings remain of questionable clinical impact. The effective overall superiority of PSI has yet to be proven.

## Figures and Tables

**Table 1:** Overview selected studies.

Author (Year)	Implant Type	Outcome Measurements	Study Design	Studied Implants		Level of Evidence	Minors Score
				OTS	PSI		
Demange (2015) [27]	UKA	Clinical and radiological (coverage, alignment)	Retrospective cohort study	Miller-Galante (Zimmer Biomet)	iUni^®^ G1 (ConforMIS)	III	16
Mayer (2020) [28]	UKA	Procedure-associated parameters, radiological (alignment), and revision rate	Retrospective cohort study	Oxford^®^ MB (Zimmer Biomet)	iUni^®^ FB (ConforMIS)	III	20
Arbab (2018) [16]	TKA	Radiological (alignment)	Retrospective cohort study	Triathlon^®^ (Stryker)	iTotal^®^ G2 CR (ConforMIS)	III	19
Buch (2019) [17]	TKA	Procedure-associated parameters, clinical, MUA, revision rate	Prospective cohort study	Columbus^®^ (B. Braun) or Vanguard^®^ (Zimmer Biomet)	iTotal^®^ G2 CR (ConforMIS)	II	20
Culler (2017) [18]	TKA	Procedure-related parameters, costs	Prospective cohort study	N/A	N/A	II	18
Ivie (2014) [19]	TKA	Radiological (alignment)	Retrospective cohort study	NK II^®^ PS (Zimmer Biomet)	iTotal^®^ G2 CR (ConforMIS)	III	18
Meheux (2019) [20]	TKA	Clinical, revision rate, radiological, procedure-associated parameters	Retrospective cohort study	GENESIS II PS (Smith&Nephew)	iTotal^®^ G2 CR (ConforMIS) and iTotal^®^ G2 plus CR (ConforMIS)	III	17
O’Connor (2019) [21]	TKA	Procedure parameters	Retrospective cohort study	N/A	iTotal^®^ (ConforMIS)	III	20
Reimann (2019) [22]	TKA	Clinical	Retrospective cohort study	Triathlon^®^ CR (Stryker)	iTotal^®^ G2 CR (ConforMIS)	III	16
Schroeder (2019) [23]	TKA	Radiological	Prospective cohort study	NexGen^®^ (Zimmer Biomet) or Vanguard^®^ (Zimmer Biomet) or SIGMA^®^ (DePuy Synthes)	iTotal^®^ CR (ConforMIS)	II	14
Schwarzkopf (2015) [24]	TKA	Clinical, procedure parameters	Retrospective cohort study	GENESIS II PS (Smith&Nephew) or SIGMA^®^ (DePuy Synthes) or P.F.C.™ SIGMA^®^ (DePuy Synthes)	iTotal^®^ G2 CR (ConforMIS)	III	15
Wheatley (2019) [25]	TKA	Clinical	Retrospective cohort study	Persona^®^ PS (Zimmer Biomet)	iTotal^®^ PS (ConforMIS)	III	18
White and Ranawat (2016) [26]	TKA	Clinical radiological	Retrospective cohort study	P.F.C.™ SIGMA^®^ PS FB cem (DePuy Synthes) or P.F.C.™ SIGMA^®^ CR RP non-cem (DePuy Synthes)	iTotal^®^ CR (ConforMIS)	III	19

Abbreviation: OTS: off-the-shelf implant, PSI: patient-specific implant, UKA: unicompartmental knee arthroplasty, TKA: total knee arthroplasty, MB: mobile bearing, FB: fixed bearing, CR: cruciate retaining, PS: posterior-stabilised, RT: rotating platform, cem: cemented, non-cem: non-cemented, N/A: not available.

**Table 2:** Patient demographics at surgery.

Author (Year)	Implant System	Number of Knees	Mean Age, Years (Range) or (SD)		Gender, Female (%)		Mean BMI, kg/m^2^, (Range) or (SD)		Mean Follow-Up Time, Months (SD)	
Demange (2015) [27]	OTS	20	56 (6.9)	ns	52.6		32.7 (7.2)		75 (20)	
	PSI	33	59 (10.9)		65.6		28.7 (5.3)		37 (8.6)	
Mayer (2020) [28]	OTS	20	61.4 (8.4)		45		31.3 (5.5)		18	
	PSI	20	62.9 (9.2)		45		29.7 (5.6)		18	
Arbab (2018) [16]	OTS	88								
	PSI	113								
Buch (2019) [17]	OTS	30	57.2 (34–67)	ns	53	ns	31 (22–38)	ns	28	
	PSI	32	57.3 (42–72)		41		33.4 (24–53)		28	
Culler (2017) [18]	OTS	122	68.3 (9.5)	ns	43.9	ns	32.3 (7.8)	ns		
	PSI	126	69.7 (8.4)		41.9		30.8 (6.5)			
Ivie (2014) [19]	OTS	100								
	PSI	100								
Meheux (2019) [20]	OTS	41	63 (10.1)				34.4 (7.1)	**	37.2 (18)	
	PSI 1	77	62.7 (8.3)				30.3 (4.5)		37.2 (18)	
	PSI 1	36	62.8 (6.7)				28.9 (5.2)		37.2 (18)	
O’Connor (2019) [21]	OTS	3695								
	PSI	739								
Reimann (2019) [22]	OTS	103	70.9 (7.1)	***	68.4	ns	31.4 (5.5)	ns	33 (7.6)	***
	PSI	125	65.5 (9.3)		63.1		30.5 (5.2)		27.5 (5.7)	
Schroeder (2019) [23]	PSI	44	70.5 (57–87)		40.9	ns	30.7 (22–49.1)			
Schwarzkopf (2015) [24]	OTS	314	65	ns	65		32.11	ns		
	PSI	307	61.4		60.2		30.85			
Wheatley (2019) [25]	OTS	124	70 (8.5)	*	64.6	ns	30.3 (8.5)		3	
	PSI	47	66.9 (7.7)		61.7		30.3 (8.5)		3	
White and Ranawat (2016) [26]	OTS, CR	42	59.8 (6.7)	ns	66.7	ns	31.8 (5.5)	ns	31.2 (8.4)	ns
	OTS, PS	11	53.9 (6.0)	*	9.1	**	34.4 (6.5)	**	27.6 (4.8)	
	PSI, CR	21	59.1 (7.4)		66.7		28.7 (4.8)		28.8 (4.8)	

Abbreviations: BMI: body mass index, kg: kilogram, m: meter, SD: standard deviation, OTS: off-the-shelf implants, PSI: patient-specific implants, CR: cruciate retaining, PS: posterior-stabilised, ns: no statistically significant difference * *p* < 0.05, ** *p* < 0.01, *** *p* < 0.001.

**Table 3:** Revisions, ROM, clinical outcomes.

Author (Year)	Implant System	Revision *n* (%)	Mean ROM (SD)		MUA *n* (%)		Mean KSS (SD) Preoperative		Mean KSS (SD) ^1^ Postoperative		FJS ^1^		WOMAC Preoperative	WOMAC ^1^ Postoperative
Demange (2015) [27]	OTS	3 (15)	pre: 122° (±9.5°) post: 127° (±7.5°)											
	PSI	2 (6.1)	pre: 125° (±8.5°) post: 125° (±6.2°)				KS: 48 (16.2)		KS: 94 (7.6)					
							*							
Mayer (2020) [28]	OTS	2 (10)												
	PSI	1 (5)												
Buch (2019) [17]	OTS	2 (6.7)	post: 144°	***	1 (3.3)	ns								
	PSI	1 (3.1)	post: 122°		2 (6.3)									
Meheux (2019) [20]	OTS	1 (2.4)	post: 122.7° (±8.2°)	ns			KS: 53.7 (10.1)	ns	KS: 91.9 (11.9)	ns				
	PSI 1	18 (23)	post: 124.2° (±6.0°)				KS: 55.5 (8.3)		KS: 94.6 (7.6)					
	PSI 2	0 (0)	post: 123.8° (±7.4°)				KS: 54.2 (6.7)		KS: 95.3 (13.3)					
Reimann (2019) [22]	OTS	1 (1.8)	pre: 110° (±13.8°) ns post: 105° (±9.2°)	ns					KS: 78.3 (13.8) FS: 68.0 (18.7)					
	PSI	1 (1.2)	pre: 110° (±15°) ns post: 105° (±9.9°)						KS: 82.4 (13.1) FS: 82.4 (13.1)	ns **				
Schwarzkopf (2015) [24]	OTS													
	PSI													
Wheatley (2019) [25]	OTS	1 (0.8)	pre: 109.4° (±9.6°) post: 119.3° (±6.1°)		2 (1.6)	ns	KS: 52.7 (10.8) FS: 56.3 (16.3)	ns	KS: 91.7 (10.2) FS: 77.6 (19.4)	ns	62.1 (25.7)	ns		
	PSI	1 (2.1)	pre: 109.3° (±9.1°) post: 118.8° (±11.0°)	ns	1 (2.1)		KS: 55.1 (12.5) FS: 51.8 (16)		KS: 91.1 (9.6) FS: 81.4 (15.3)		56.0 (26.9)			
White and Ranawat (2016) [26]	OTS, CR	0 (0)	pre: 111° (12°) post: 118° (8°)	**	0		KS: 45.7 (9) FS: 51.1 (10.4)		KS: 95.5 (7.1) FS: 88.9 (13.8)	ns			TS: 52.4 (12.8) PS: 11.1 (2.8) SS: 5.1 (1.4) FS: 36.2 (9.7)	TS: 7.8 (8.4) * PS: 1.2 (2.5) SS: 1.3 (2.1) FS: 5.2 (5.8)
	OTS, PS	0 (0)	pre: 114° (10°) post: 120° (4°)		0		KS: 45.2 (9) FS: 54.1 (13.2)		KS: 97.3 (3.9) FS: 96.4 (5)	*			TS: 41.3 (9.6) PS: 7.8 (1.9) SS: 3.4 (1.6) FS: 30.1 (7.36)	TS:15.4 (18.3) PS:2.8 (4) SS: 2.2 (2.3) FS: 10.4 (12.9)
	PSI, CR	1 (4.8)	pre: 120° (12°) post: 115° (10°)	ns	6 (28.6)		KS: 53.6 (8.3) FS: 54 (12.2)	** ns	KS: 85.4 (15.5) FS: 86 (14.8)	**			TS: 51.4 (17) PS: 11.5 (3.9) SS: 4.6 (2.5) FS: 35.3 (12.3)	TS: 23.4 (23.1) * PS: 4.8 (5.3) SS: 3 (2.4) FS: 15.2 (16.3)

Abbreviations: ROM: range of motion, MUA: manipulation under anaesthesia, KSS: Knee Society Score, KS: knee score, FS: function score, FJS: Forgotten Joint Score, WOMAC: Western Ontario and McMaster Universities Arthritis Index, TS: total score, PS: pain score, SS: stiffness score, FS: function score, SD: standard deviation, OTS: off-the-shelf implants, PSI: patient-specific implants, CR: cruciate-retaining, PS: posterior-stabilised, pre: preoperative, post: postoperative, ns: no statistically significant difference * *p* < 0.05, ** *p* < 0.01, *** *p* < 0.001. ^1^ clinical outcome scores at last follow up.

**Table 4:** Radiological outcome.

Author (Year)	Implant System	Mean FFC (SD)		Mean FTC (SD)		Mean Tibial Slope (SD)		Mean HKA ^1^ (SD) or (Range)				> ±3° HKA Outliers	Femorotibial Angle ^1^				Optimal Tibial Fit ^a^ Resp. Relative Undercoverage ^b^	
								Pre-Op		Post-Op			Pre-Op		Post-Op			
Demange (2015) [27]	OTS																21.1% ^a^	
	PSI							3.3° (4.9°) (−5.4°–+8.5°)		−0.9° (3.8°) (−8.0°–3.4°)							75.8% ^a^	
Arbab (2018) [16]	OTS, CR							8.2° (−18.2°–+15.7°) median 5.6°		2.3° (−10.1°–+12.5°) median 1.7°		26%						
	PSI, CR							9.0° (−27.3°–+18.9) median 5.7°		3.2° (−7.6°–+8.4°) median 0.7°		16%						
Ivie (2014) [19]	OTS	88.32° (1.51°)	*	87.81 (1.54)	ns	87.12° (1.73°)	ns			1.68° (3.65°)	**	43.1%						
	PSI	87.37° (3.87°)		87.71° (1.44°)		86.42° (2.61°)				−0.47° (3.15°)		29.6%						
Meheux (2019) [20]	OTS			88.54° (1.5°)		4.00° (2.5°)		−3.32° (5.2°)	ns	−3.32° (5.2°)	ns				2.29° (3.8°)			
	PSI 1			91.08° (1.9°)		6.40° (2.9°)		−3.97° (3.5°)		−1.34° (4.6°)					4.09° (2.7°)			
	PSI 2			89.89° (1.0°)		5.53° (3.9°)		−3.89° (3.46°)		−0.35° (1.8°)					4.1° (3°)			
Schroeder (2019) [23]	OTS 1																23% ^a + b^	***
	OTS 2																25% ^a + b^	
	OTS 3																34% ^a + b^	
	PSI																80% ^a + b^	
White and Ranawat (2016) [26]	OTS, CR					5° (1°)							−4° (3°)	ns	2°	ns		
	OTS, PS					4° (1°)							−1° (7°)		2°			
	PSI, CR					5° (1°)							−3° (4°)		2°			

Abbreviations: FFC: frontal femoral component angle, FTC: frontal tibial component angle, pre-op: preoperative, post-op: postoperative, HKA: hip–knee–ankle, SD: standard deviation, OTS: off-the-shelf implants, PSI: patient-specific implants, CR: cruciate retaining, PS: posterior-stabilised, ns: no statistically significant difference. * *p* < 0.05, ** *p* < 0.01, *** *p* < 0.001. ^1^ varus knees were recorded as negative values and valgus as positive ^a^ 1 mm implant overhang to 1 mm tibial bone undercoverage ^b^ 1–3 mm tibial undercoverage.

## Data Availability

Not applicable.
